# Musculoskeletal infections associated with *Nocardia* species: a case series

**DOI:** 10.5194/jbji-9-207-2024

**Published:** 2024-09-24

**Authors:** Ryan B. Khodadadi, Jack W. McHugh, Supavit Chesdachai, Nancy L. Wengenack, Wendelyn Bosch, Maria Teresa Seville, Douglas R. Osmon, Elena Beam, Zachary A. Yetmar

**Affiliations:** 1 Division of Public Health, Infectious Diseases and Occupational Medicine, Department of Medicine, Mayo Clinic, Rochester, Minnesota, USA; 2 Division of Clinical Microbiology, Mayo Clinic, Rochester, Minnesota, USA; 3 Division of Infectious Diseases, Mayo Clinic, Jacksonville, Florida, USA; 4 Division of Infectious Diseases, Mayo Clinic, Phoenix, Arizona, USA; 5 Department of Orthopedic Surgery, Mayo Clinic, Rochester, Minnesota, USA; 6 Department of Infectious Disease, Cleveland Clinic, Cleveland, Ohio, USA

## Abstract

**Background**: *Nocardia* is an uncommon pathogen that has been reported to infect musculoskeletal structures. However, studies are largely limited to case reports, and little is known regarding management and outcomes of these infections. **Methods**: We performed a multicenter retrospective cohort study of adults with culture-confirmed musculoskeletal *Nocardia* infections at three Mayo Clinic centers in Arizona, Florida, and Minnesota from November 2011 through April 2022. **Results**: Nine cases of *Nocardia* musculoskeletal infection were identified. Seven (78 %) occurred in men, and the median age was 57.3 years (range 32.6–79.0). Specific infections included native joint septic arthritis with or without associated osteomyelitis ( N=3 ), hardware-associated infection ( N=1 ), sternal osteomyelitis ( N=1 ), pyomyositis ( N=2 ), bursitis ( N=1 ), and tenosynovitis ( N=1 ). Three cases (33 %) were associated with disseminated disease, all three occurring in solid organ transplant recipients. Surgical intervention was performed in all but the bursitis case. Length of treatment varied from 21 d for tenosynovitis to 467 d for osteomyelitis. The 1-year mortality was 22 %, and all fatal cases involved disseminated disease. **Conclusion**: Patients with localized nocardiosis affecting musculoskeletal structures generally have good outcomes, as opposed to those with disseminated infection. Management often required operative intervention, with one patient experiencing recurrence within 1 year.

## 1 Introduction

*Nocardia* is a genus of aerobic filamentous Gram-positive bacteria that can cause infections in both immunocompetent and immunocompromised individuals (Traxler et al., 2022; Wilson, 2012). Clinical manifestations of nocardiosis are diverse, ranging from localized cutaneous lesions to disseminated infection with multiorgan involvement (Wilson, 2012). These infections can be challenging to diagnose and manage due to their indolent nature and the organism's potential resistance to various antimicrobial agents (Wilson, 2012).

*Nocardia* musculoskeletal infections including native joint septic arthritis, prosthetic joint infection, osteomyelitis, and tenosynovitis are rare, and there are sparse data describing associated clinical characteristics, management, and outcomes, with studies to date confined to case reports and small case series (Chaussade et al., 2015; Takamatsu et al., 2022). Given the limited information in the literature, we sought to review our institutional experience over the past decade with *Nocardia* musculoskeletal infections, outlining demographic, clinical, diagnostic, and treatment strategies as well as outcomes.

## 2 Materials and methods

### 2.1 Study design

We performed a multicenter, retrospective cohort study of adults ( ≥18 years) with musculoskeletal infection from culture-confirmed *Nocardia* species obtained from clinical specimens at three Mayo Clinic centers in Arizona, Florida, and Minnesota from November 2011 to April 2022. Patients with culture growth of a *Nocardia* species were identified through our microbiology laboratory's internal registry. Patients were then manually screened for clinical infection as part of a separate study, and those with infection involving musculoskeletal structures were included in this study (Yetmar et al., 2023). Patients with a clinical syndrome compatible with musculoskeletal infection were identified for further analysis. Patients were excluded if they lacked clinical signs, symptoms, and/or radiographic changes consistent with infection; their age was less than 18 years on the date of diagnosis; or they lacked research authorization per Minnesota state statute (Minnesota Statutes, 2023).

Following the preliminary screening process, data including demographics; comorbidities included in the Charlson Comorbidity Index (CCI); race; ethnicity; and further details regarding lung disease, immunocompromised states including solid organ transplantation (SOT), hematopoietic stem cell transplant (HSCT), and immunosuppressant use were extracted from the electronic medical record. In addition, clinical, microbiologic, and treatment characteristics were collected manually from the electronic medical record and managed using REDCap electronic data capture tools (Harris et al., 2019, 2009). The clinical microbiology laboratory at Mayo Clinic in Rochester, Minnesota, received specimens for cultures, identification, and susceptibility testing from Mayo Clinic sites. Antimicrobial susceptibility testing was interpreted per guidelines of the Clinical and Laboratory Standards Institute (Clinical and Laboratory Standards Institute, 2018a, b). Additional information regarding *Nocardia* species identification and susceptibility testing for isolates in our reference microbiology laboratory at Mayo Clinic is described in a previous study (Yetmar et al., 2024). This study was reviewed and granted exempt status by our internal institutional review board (no. 22-008346).

### 2.2 Definitions

Musculoskeletal infection attributable to *Nocardia* species was defined as growth of *Nocardia* in culture with compatible signs, symptoms, and findings indicative of native or prosthetic musculoskeletal clinical infection. Date of diagnosis was defined as the date of the first culture acquisition that yielded *Nocardia* species. Localized disease was defined as isolation of *Nocardia* from the primary site of infection without evidence of additional sites of involvement. Disseminated infection was defined as the involvement of at least two non-contiguous organs. Immunocompromised status was defined as receipt of at least 20 mg d^-1^ of prednisone equivalent corticosteroid, receipt of other immunosuppressive medication, or HSCT within the preceding 100 d. Immunosuppressant use was assessed within the preceding 28 d of *Nocardia* diagnosis. Similarly, corticosteroid dosing was the most recent recorded dose in the 28 d prior to infection, converted to equivalent dosing of prednisone. Trimethoprim–sulfamethoxazole (TMP-SMX) prophylaxis was assessed on the date of initial presentation. Laboratory values were assessed on the date of diagnosis or the most recent prior measurement. Combination therapy was defined as receipt of at least two initial antibiotic agents. Active antibiotic agents were those that were initially used and later tested to be susceptible. Definitive antimicrobial therapy was defined as tailored antimicrobial therapy utilized for over half of a patient's treatment course. Progression was defined as clinical, symptomatic, or radiographic worsening of disease despite effective antimicrobial therapy for *Nocardia* musculoskeletal infection.

### 2.3 Statistical analysis

Descriptive statistics in this series are described as either a median value (range) for continuous variables or as a number (percentage) for categorical variables. The CCI was calculated as previously described (Charlson et al., 1994). All analyses were performed using R version 4.3.1 (R Foundation for Statistical Computing, Vienna, Austria).

## 3 Results

From 511 patients with culture growth of *Nocardia* from a clinical specimen, 9 patients were diagnosed with *Nocardia* musculoskeletal infection. The median age was 57.3 years (range 32.6–79.0), with most patients being male ( N=7 , 78 %) and of white race ( N=7 , 78 %) and with a median CCI of 2.0 (range 0–6). The most common comorbidities observed were chronic kidney disease ( N=4 , 44 %), diabetes mellitus ( N=3 , 33 %), and prior SOT ( N=4 , 44 %), all of which were kidney transplants. Five patients were considered immunocompromised: four were receiving prednisone, tacrolimus, and mycophenolate following SOT, and one patient was receiving chronic prednisone for management of ulcerative colitis. Six patients had localized infection, while three patients presented with disseminated disease; all three with disseminated infection had a history of SOT. The type of musculoskeletal infection varied and included native joint septic arthritis with or without associated osteomyelitis ( N=3 , 33 %), ankle hardware-associated infection ( N=1 , 11 %), sternal osteomyelitis ( N=1 , 11 %), pyomyositis ( N=2 , 22 %), bursitis ( N=1 , 11 %), and tenosynovitis ( N=1 , 11 %). With respect to the hardware-associated infections, one patient developed infection of sternotomy wires 2 months following repair of an aortic aneurysm; the other developed purulent drainage at the site of pin placement 2 months following closed reduction and external fixation of an ankle fracture. There were no cases of prosthetic joint infection. The most common *Nocardia* species isolated included *N. cyriacigeorgica* ( N=2 , 22 %), *N. nova* ( N=2 , 22 %), and *N. farcinica* ( N=2 , 22 %). Additional cohort characteristics and case details are outlined in Table 1.

**Table 1:** Case details of nine patients with *Nocardia* musculoskeletal infection.

	Sex	Age	CCI	History of SOT	IS usewithin 28 d	*Nocardia* sp.	Infection site(s)	Mechanism ofinfection	Disseminated infection	Procedure type	Definitive antibiotic treatment	Treatment duration (days)	Progression	Comments
Case 1	Female	60	0	No	No	*N. abscessus*	First nativemetatarsophalangeal joint septic arthritis and associated OM	Post-operative	No	Arthrotomy and debridement	TMP-SMX	89	No	Infection developedpost-bunionectomy
Case 2	Female	32	2	Yes	Yes	*N. cyriacigeorgica*	Hardware-associated left-ankle septic arthritis with associated tibial OM	Post-operative	No	Debridement and hardware removal	TMP-SMX Minocycline	371	No	Initial treatment failure after stopping TMP-SMX at 3 months
Case 3	Male	65	4	No	Yes	*N. cyriacigeorgica*	Left deltoid pyomyositis	Post-corticosteroid injection	No	Incision and drainage	CeftriaxoneTMP-SMX	190	No	Intravenous therapy for first 6 weeks
Case 4	Male	46	0	No	No	*N. farcinica*	Sternal osteomyelitis	Post-operative	No	Debridement and wire removal with flap placement	Meropenem TMP-SMX Ciprofloxacin	467	Yes	Initial treatment failure on TMP-SMX monotherapy
Case 5	Male	42	0	No	No	*N. nova*	Left fifth-finger flexor tenosynovitis	Traumatic	No	Incision and drainage	TMP-SMX	21	No	Traumatic injury.Polymicrobial flexor tenosynovitis.
Case 6	Male	57	2	No	No	*N. nova*	Left prepatellar bursitis	Cryptogenic	No	No procedure	TMP-SMX	182	No	No risk factors or clear history of trauma
Case 7	Male	79	6	Yes	Yes	*N. pseudobrasiliensis*	Left native knee joint septic arthritis Cutaneous, pulmonary	Hematogenous	Yes	Arthroscopic irrigation and debridement left knee	Azithromycin Linezolid Ciprofloxacin	193	No	Received 1 week of intravenous therapy with ceftriaxone
Case 8	Male	41	4	Yes	Yes	*N. otitidiscaviarum*	Left nativewrist jointseptic arthritis with associated osteomyelitis Pulmonary, brain, bacteremia	Hematogenous	Yes	Irrigation and debridement	Imipenem TMP-SMX Minocycline Clarithromycin Moxifloxacin	314	No	Received 8 weeks of imipenem. Disease was stable at 6-month follow-up. Cause of death unclear.
Case 9	Male	68	5	Yes	Yes	*N. farcinica*	Left-shoulderpyomyositis, pulmonary infection, brain abscesses	Hematogenous	Yes	Irrigation and debridement	Imipenem Moxifloxacin	40	No	Died after leaving a skilled nursing facility against medical advice

CCI: Charlson Comorbidity Index. SOT: solid organ transplant. IS: immunosuppressant. TMP-SMX: trimethoprim–sulfamethoxazole. OM: osteomyelitis.

Antimicrobial susceptibility results are shown in Table 2, which were available for all nine isolates. All isolates were susceptible to amikacin ( N=9 , 100 %) and linezolid ( N=9 , 100 %), and most isolates were susceptible to TMP-SMX ( N=7 , 78 %). Otherwise, susceptibility to other antimicrobial agents varied, with imipenem being the most effective among beta-lactams. For treatment, most patients received TMP-SMX ( N=7 , 78 %), followed by imipenem ( N=4 , 44 %) and linezolid ( N=3 , 33 %). The median number of initial agents was two (range one to three), with a median length of therapy of 190 d (range 21–467). Of the four cases occurring in SOT, mycophenolate was held in three cases where there was evidence of disseminated disease, and tacrolimus and prednisone were continued. In combination with antimicrobial therapy, surgical intervention was performed in all cases but one for surgical source control. For localized infection, a single surgery was effective for clinical cure in four of six cases (67 %). The 1-year mortality was 22 %, occurring at 42 and 327 d after *Nocardia* diagnosis, respectively. Median post-treatment follow-up was 512 d (range: 160–3451). Only two patients received secondary prophylaxis with either TMP-SMX or doxycycline for confirmed susceptible isolates.

**Table 2:** Antimicrobial susceptibility results.

	Number susceptible
	( N=9 )
Amikacin	9 (100 %)
Amoxicillin–clavulanate	3 (33 %)
Ceftriaxone	3 (33 %)
Ciprofloxacin	3 (33 %)
Clarithromycin	4 (44 %)
Imipenem	6 (67 %)
Linezolid	9 (100 %)
Minocycline	1 (11 %)
Moxifloxacin	4 (44 %)
Tobramycin	4 (44 %)
Trimethoprim–sulfamethoxazole	7 (78 %)

Following initial treatment, progression was observed in one case of TMP-SMX-susceptible *N. farcinica* sternal osteomyelitis. This occurred on TMP-SMX monotherapy, likely due to incomplete initial surgical debridement, as the patient ultimately required serial debridement procedures followed by sternal wire removal and flap placement. In the case of hardware-associated left-ankle septic arthritis, the patient developed purulent drainage from external pins 3 months post-closed reduction of a left calcaneal fracture. Hardware was removed, and 12 months of treatment was recommended. TMP-SMX monotherapy was abruptly discontinued by the patient after 3 months with recrudescence of infection due to incomplete treatment confirmed on operative cultures at the time of repeat surgery. Following additional operative intervention to achieve surgical source control and tailoring of empiric therapy to *N. abscessus* susceptibilities, the patient completed 281 additional days of treatment with TMP-SMX and minocycline.

## 4 Discussion

Musculoskeletal infections caused by *Nocardia* species pose significant challenges in clinical management due to their rare occurrence, variable clinical presentations, and the need for prolonged antibiotic therapy. In this study, we reviewed our institutional experience and investigated clinical characteristics, management strategies, and outcomes of musculoskeletal *Nocardia* infections to enhance our understanding and further inform clinical practice of this rare condition.

Our findings revealed a low incidence of musculoskeletal *Nocardia* infections amongst all cases of nocardiosis at our institution over the past decade, consistent with previous reports (Takamatsu et al., 2022; Chaussade et al., 2015). In 2022, a literature review identified 37 reported cases of septic arthritis from *Nocardia* species, 5 of which were prosthetic joint infections (Fazili et al., 2022). Other *Nocardia* musculoskeletal infections such as osteomyelitis, pyomyositis, and tenosynovitis have been confined to case reports. The rarity of these infections emphasizes the importance of a high index of suspicion, particularly in patients with underlying immunocompromising conditions or a recent history of trauma or surgery.

Clinical manifestations of musculoskeletal *Nocardia* infection varied widely, ranging from localized infection secondary to trauma to deeper involvement of musculoskeletal structures as a consequence of dissemination in immunocompromised hosts (Wilson, 2012). Optimal management of musculoskeletal *Nocardia* infections requires a multidisciplinary approach, involving orthopedic surgeons and infectious diseases specialists (Wilson, 2012). Surgical intervention, such as drainage of abscesses and debridement of infected tissues, is often necessary, particularly in cases of extensive involvement or implant-associated infections. Indeed, nearly all patients in our cohort required a surgical procedure, with one requiring further surgeries due to progression after an incomplete debridement. Our study reaffirms the importance of timely and aggressive surgical debridement to control these infections and prevent disease progression.

In combination with surgical source control, prolonged antimicrobial therapy remains a cornerstone of treatment for musculoskeletal *Nocardia* infections. However, selecting the most appropriate antibiotic regimen upfront remains challenging due to the limited clinical data on musculoskeletal *Nocardia*, bone penetration of available antimicrobials, long turnaround times for species identification, and variable susceptibility patterns among *Nocardia* species. Our study identified TMP-SMX as the most commonly used antibiotic agent, either as monotherapy or in combination with other antimicrobials depending on extent of involvement.

The optimal duration of treatment for musculoskeletal *Nocardia* infections is overall poorly understood, and individualized treatment takes into consideration a multitude of factors including the immune status of the affected patient, extent of involvement, and whether surgical source control has been effectively achieved as well as response to initial therapy (Restrepo and Clark, 2019; Wilson, 2012). While treatment duration for nocardiosis remains controversial, some studies have suggested a longer duration of treatment may be associated with fewer episodes of post-treatment recurrence (Yetmar et al., 2024; Tashiro et al., 2018). This risk likely is affected by the site of infection, and most patients included in this cohort of musculoskeletal infections were treated with prolonged antibiotics. One patient did have an early recurrence after a relatively brief course of treatment (90 d) though ultimately had a good outcome after restarting treatment for a longer period. Two patients also utilized post-treatment secondary prophylaxis, though data are limited overall regarding the use of prophylaxis for nocardiosis (Yetmar et al., 2024; Passerini et al., 2024). Long-term antibiotic suppression is commonly utilized in patients with musculoskeletal infection, particularly those with retained prosthetic material (Cortes-Penfield et al., 2023). Further study is needed to evaluate treatment duration and prophylaxis for nocardiosis, though close follow-up and clinical assessment for evaluation of treatment response and complications during and after therapy are crucial.

Favorable clinical responses were observed in the majority of patients following appropriate surgical and medical management. However, a subset of patients, in particular one SOT patient, experienced treatment failure with progression, highlighting the importance of long-term follow-up and surveillance to determine if failure is related to antibiotic therapy, lack of surgical source control, and profound immunosuppression (Restrepo and Clark, 2019). Further research is warranted to elucidate the prognostic factors associated with poor outcomes and refine treatment algorithms for musculoskeletal *Nocardia* infections.

Strengths of this study include the number of included cases, as most prior studies are case reports. We also had susceptibility data for all isolates as well as robust clinical, diagnostic, and treatment details available in the electronic medical record at our institution. However, there are several limitations worth noting. While large for this topic, there were still relatively few cases, which may limit the generalizability of this study's findings and precluded more formal statistical analyses. Further, the considerable diversity in underlying clinical syndromes precludes the formulation of overarching conclusions. Additionally, this study was retrospective and observational in nature and prone to unmeasured sources of bias. In addition, there were only a few patients with disseminated disease, and overall, there was a lack of uniform follow-up.

## 5 Conclusion

In conclusion, musculoskeletal *Nocardia* infections represent a rare but clinically significant entity that requires prompt recognition and aggressive management. Our study provides valuable insights into the epidemiology, clinical characteristics, and treatment outcomes of these infections, guiding clinicians in their approach to diagnosis and management. Collaborative efforts across specialties, continued surveillance of antimicrobial resistance patterns, and further research are crucial for improving patient outcomes and reducing the burden of musculoskeletal *Nocardia* infections.

## Data Availability

The dataset is available upon request.
